# The comparison of the effect of non-pharmacological pain relief and pharmacological analgesia with remifentanil on fear of childbirth and postpartum depression: a randomized controlled clinical trial

**DOI:** 10.1186/s12884-024-06270-z

**Published:** 2024-04-23

**Authors:** Parinaz masroor, Esmat Mehrabi, Roghaiyeh Nourizadeh, Hojjat Pourfathi, Mohammad Asghari-Jafarabadi

**Affiliations:** 1 Midwifery Department, Faculty of Nursing and Midwifery, Students’ Research Committee, Tabriz University of Medical Sciences, Tabriz, Iran; 2 Department of Midwifery, Faculty of Nursing and Midwifery, Tabriz University of Medical Sciences, Tabriz, Iran; 3 Department of Anesthesiology and Pain Management, Faculty of Medicine, Tabriz University of Medical Sciences, Tabriz, Iran; 4 Cabrini Research, Cabrini Health, Malvern, VIC 3144 Australia; 5 School of Public Health and Preventative Medicine, Faculty of Medicine, Nursing and Health Sciences, Monash University, Melbourne, VIC 3004 Australia; 6 Road Traffic Injury Research Center, Tabriz University of Medical Sciences, Tabriz, Iran

**Keywords:** Fear of childbirth, Non-pharmacological pain relief, Labor pain, Postpartum depression, Remifentanil

## Abstract

**Introduction:**

Childbirth may be associated with psychological, social, and emotional effects and provide the background for women’s health or illness throughout their life. This research aimed at comparing the impact of non-pharmacological pain relief and pharmacological analgesia with remifentanil on childbirth fear and postpartum depression.

**Materials and method:**

This randomized clinical trial with two parallel arms was conducted on 66 women with term pregnancy referred to Taleghani Hospital in Tabriz for vaginal delivery during September 2022 to September 2023. First, all of the eligible participants were selected through Convenience Sampling. Then, they were randomly assigned into two groups of pharmacological analgesia with remifentanil and non-pharmacological analgesia with a ratio of 1:1 using stratified block randomization based on the number of births. Before the intervention, fear of childbirth (FOC) was measured using Delivery Fear Scale (DFS) between 4 and 6 cm cervical dilatation. Pain and fear during labor in dilatation of 8 cm were measured in both groups using VAS and DFS. After delivery, FOC was assessed using Delivery Fear Scale (W DEQ Version B) and postpartum depression using the Edinburgh’s postpartum depression scale (EPDS). Significance level was considered 0.05. Mean difference (MD) was compared with Independent T-test and ANCOVA pre and post intervention.

**Results:**

The mean score of FOC in the non-pharmacological analgesia group was significantly lower than that in the pharmacological analgesia group after the intervention by controlling the effect of the baseline score (MD: -6.33, 95%, Confidence Interval (CI): -12.79 to -0.12, *p* = 0.04). In the postpartum period, the mean score of FOC in the non-pharmacological analgesia group was significantly lower than that in the pharmacological analgesia group after controlling the effect of the baseline score (MD: -21.89; 95% CI: -35.12 to -8.66; *p* = 0.002). The mean score of postpartum depression in the non-pharmacological analgesia group was significantly lower than that in the pharmacological analgesia group (MD: -1.93, 95% CI: -3.48 to -0.37, *p* = 0.01). Trial registration: Iranian Registry of Clinical Trials (IRCT): IRCT20170506033834N10. Date of registration: 05/07/2022 Date of first registration: 05/07/2022. URL: https://www.irct.ir/trial/61030; Date of recruitment start date05/07/2022.

**Conclusion:**

The study results indicated a reduction in FOC and postpartum depression among parturient women receiving non-pharmacological strategies with active participation in childbirth compared to women receiving pharmacological analgesia. Owing to the possible side effects of pharmacological methods for mother and fetus, non-pharmacological strategies with active participation of the mother in childbirth are recommended to reduce the FOC and postpartum depression.

## Introduction

Fear of childbirth (FOC) is a problem that nulliparous, and multiparous women brave with health consequences and implications for labor and the puerperium [1, 2]. An American surgeon, Jim Capa, (1885), in an interview with the New England Journal of Medicine stated that labor pain makes the mother reluctant to give birth again due to the FOC. Since then, the medical circle and society have paid special attention to the treatment of pain during childbirth. FOC is described as the negative feelings toward childbirth and pregnancy. Some factors, such as fear of pain, death, and unexpected problems, poor self-efficacy, worry about sexual problems after childbirth and baby’s health are regarded as the main reasons for childbirth fear [3]. FOC ranges from severe to rational fear and most women, especially primiparous women, experience a rational fear due to being unfamiliar with the birthing process, which is naturally controlled during pregnancy and delivery [2]. FOC entails stress, nightmares, and physical symptoms. FOC can involve consequences, such as postpartum depression, tendency to have an abortion, post-traumatic stress disorder [4], premature birth, low birth weight, intrauterine growth restriction of the fetus [5], abnormal fetal heart rate, low Apgar score of the baby, and increased mortality during birth [6]. One of the main consequences of the fear of labor pain is increasing request for Caesarean section (CS) [4]. Fear during labor causes a vicious circle of contractions and medical interventions and increases the possibility of experiencing a difficult delivery. Further, fear makes women experience more pain during labor, which leads to negative experience of childbirth. Further, women with severe pain and fear during pregnancy and labor experience emotional imbalance after childbirth [7].

Postpartum period is regarded as one of the challenging periods for mothers and postpartum depression increases throughout a woman’s life. Postpartum depression is considered as a common mental and social health problem and a widespread complication of childbearing, usually occurring within 4 to 6 weeks after delivery, which may last for several months or even a year. Moreover, up to 50% of women experience depression recurrence in subsequent pregnancies [8]. Postpartum depression is defined as depressive symptoms, such as low mood, loss of pleasure, decreased energy and activity, functional impairment, low self-esteem, and suicidal thoughts or actions occurring in the first year after delivery. Empirical evidence indicated that postpartum depression is related to mother-infant bonding disorder, child abuse, child neglect, substance abuse, and self-harm. In addition, maternal depression is associated with poor weight gain, impaired cognitive and motor development in infants, and early cessation of breastfeeding due to insufficient breast milk, accounting for about 22% of maternal deaths [9]. Labor analgesia interventions may be associated with reducing the risk of postpartum depression.

Labor pain management is not only a critical concern for expectant mothers, but also a major challenge in modern medicine. Currently, a wide range of pharmacological and non-pharmacological analgesia techniques are available for pregnant women. Non- pharmacological techniques include water birth, transcutaneous electrical nerve stimulation (TENS), aromatherapy, acupuncture, massage and breathing techniques, the presence of a supportive person during labor, and upright positions during labor. Pharmacological techniques include inhalational analgesia, opioid and non-opioid drugs, epidural analgesia, and anesthetic nerve blocks [10]. Remifentanil is an opioid and fast-acting medicine with peak effect after intravenous administration in 60–90 s, which has attracted the attention of researchers due to its minimal effect on the fetus [11].

Owing to the technological advancement and continuous improvement of painless delivery techniques in the mid-1990s, painless delivery has become a new trend of care, which is selected by increased number of women. Although most hospitals in our country currently support painless delivery methods, there are still hospitals that are reluctant to accept painless delivery due to limited conditions, pregnant women’s own conditions, and a misunderstanding of anesthesia. For this reason, women still have a high desire to CS to get rid of the labor pain. However, the high rate of CS can impose heavy costs on the insurance and ultimately, the economy of the society and increase the risk of mortality and complications for both baby and mother. Since the fear of labor pain is often regarded as the most common cause of elective cesarean section, implementing effective and safe pain relief methods in the course of vaginal delivery in maternity hospitals can reduce CS and its resultant complications. Given that safe, low-cost, and applicable methods should always be adopted in alleviating labor pain and owing to the little information available in this area and lack of the comparison studies in relation to type of pain relief and fear of childbirth and postpartum depression and also Considering the long-term persistence of childbirth experience in the mind of women and the relationship between birth, fear, and postpartum depression, the present study aimed to evaluate the effect of non-pharmacological analgesia and pharmacological analgesia with remifentanil on FOC and postpartum depression.

### Assessed outcomes

The primary outcomes of this study were FOC and postpartum depression and secondary outcomes were labor pain, Apgar score of the baby, and frequency of CS.

## Method

### Study design and participants

This randomized clinical trial was performed on 66 pregnant women with a gestational age of 37–42 weeks referred to Taleghani Education and Treatment Hospital in Tabriz. The inclusion criteria were all literate women aged 18 years and older with a gestational age of 37–42 weeks, who were going to give their first or second vaginal delivery in Taleghani Hospital during September 2022 to September 2023.

The exclusion criteria included non-cephalic presentation, indication for CS such as abnormal presentation, placenta previa, etc., obstetric problems such as placenta previa, vaginal delivery after CS, placental abruption, and preeclampsia, high-risk pregnancies such as diabetes, cardiovascular disease, etc., willingness to use other analgesia methods, history of participating in physiological childbirth classes, body mass index of 35 or above, hospitalization in dilation after 6 cm and unplanned pregnancy, having a history of depression based on the medical profile or the use of anti-anxiety and depression drugs, and the occurrence of trauma during the last 6 months in the family such as the death of a close relative and divorce.

The sample size was calculated based on the scores of both FOC and depression using G-Power software, assuming a 15% reduction in the scores of both variables, with a test power of 95% and a 10% sample loss. Based on FOC data of the study of Khorsandi et al. [12], m_1_ = 39.35 (The mean score of FOC before the intervention), m_2_ = 33.45 (The mean score of FOC after the intervention), sd_1_ = sd_2_ = 6.96, α = 0.05, the sample size was calculated to be 31 and based on depression data of the study of Abdollahpour et al., m_1_ = 7.8 (The mean score of depression before the intervention), m_2_ = 4.9 (The mean score of depression after intervention), sd_1_ = 3.65, sd_2_ = 2.71, α = 0.05, the sample size was estimated to be 21 [13]. The sample size obtained based on the FOC was more than that of the other variables,, the final sample size was calculated as 33 considering a 10% attrition.

### Sampling

The ethics committee of Tabriz University of Medical Sciences approved this study (IR.TBZMED.REC.1401.231). After registering the study in the Iranian Registry of Clinical Trials (IRCT), (IRCT20170506033834N10), the researcher selected the sample among all eligible women referred to Taleghani Hospital using convenient sampling method. The participants completed the written informed consent form to participate in the study.

### Randomization and allocation concealment

The participants were assigned into two groups, including one with pharmacological analgesia (receiving remifentanil during active phases of child birth and after 6 cm cervix dilatation) and the other with non-pharmacological analgesia (back massage, lukewarm water abdominal shower, pressure on the sacrum, breathing techniques, and upright positions) with a ratio of 1:1 based on the stratified block randomization based on the number of births using Random Allocation Software (RAS) with block size of 4 and 6. The type of intervention was written on paper and placed in sequentially numbered opaque envelopes to conceal the allocation. After signing the written informed consent form, the corresponding envelope was opened and the intervention was implemented. Given that the researcher did not know the type of group until opening the envelope, the study is one-sided blind.

### Intervention and follow-up

For the pharmacological analgesia group, the remifentanil infusion was performed in 4–6 cm cervix dilatation by an anesthesiologist using a continuous intravenous (IV) infusion pump at a dose of 0.5 µg/kg/min until the complete cervical dilatation. It is worth mentioning that pharmacological analgesia (including remifentanil, pethidine, and hyoscine) is proposed routinely to the parturient women to reduce labor pain in Taleghani Hospital. In the active phase of labor in the dilatation of 4–6 cm, back massage, warm water abdominal shower, pressure on the sacrum, breathing techniques, and upright positions were used for the participants in the non-pharmacological analgesia group by first author who was experienced in physiologic birth. In the non-pharmacological analgesic group, the participants were encouraged to actively cooperate in childbirth (movement, breathing, abdominal showering, etc. during labor). While in the pharmacological analgesia group, mother could not cooperate in her childbirth process and was inactive during labor due to the effect of the drug. Before the intervention, socio-demographic and obstetric characteristics questionnaire was completed and pain intensity in cervix dilatation (during childbirth) of 4–6 cm were assessed using the Visual Analog Scale (VAS) and FOC using the Delivery Fear Scale (DFS). All non-pharmacological methods were suggested to the mothers to choose each one if she wished. In general, almost most of the mothers in the non- pharmacological group found a combination of the mentioned interventions) back massage, warm water abdominal shower, pressure on the sacrum, breathing techniques, and upright positions (in the active phase of labor in the dilatation of 4–6 cm. The first author as a midwife attended in the delivery room during the childbirth process of all the participants in both groups to avoid confounding effect of the midwife’s presence in the study.

Labor pain and FOC were measured again in cervix dilatation of 8 cm (during childbirth) in both groups. Delivery Fear Scale (W DEQ Version B) and Edinburgh’s postpartum depression scale (EPDS) were used to assess FOC and postpartum depression one month after delivery through interview.

**Fig. 1:**
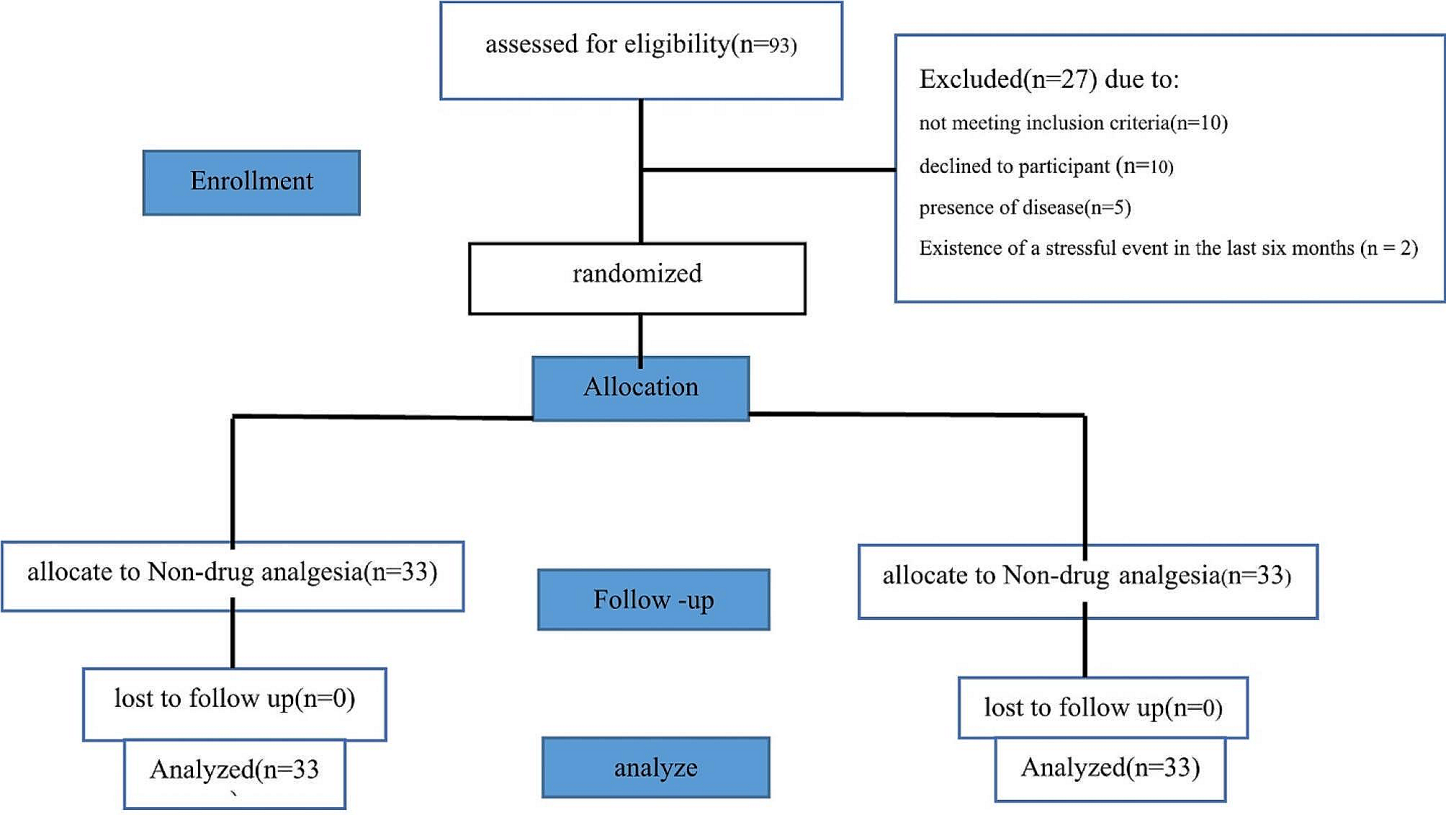
Flow diagram of study

The researcher evaluated 93 pregnant mothers, of which seven women with high-risk pregnancy (diabetes, cardiovascular disease, and abnormal fetus), two with a recent stressful event, and ten with unwillingness to participate in the study were excluded and 66 eligible women were selected as sample. There was no loss to follow-up and all mothers were followed up one month after delivery (Fig. 1).

### Scales and data collection

Data were collected using the questionnaires of socio-demographic and obstetric characteristics, Delivery Fear Scale, Wijma Delivery Expectancy/Experience Questionnaire (WDEQVersion B), postpartum depression questionnaire, and Visual Analogue Scale.

The socio-demographic questionnaire contained items about age, education, occupation, family income level, induction or augmentation of labor by Oxytocin, use nipple stimulation during childbirth, receiving pharmacological analgesia with hyoscine or remifentanil, gender of newborn, etc. The content validity of this questionnaire was assessed and confirmed by an expert panel, including ten experts in the fields of midwifery, reproductive health, obstetrics, and gynecology.

The fear during labor was measured using Delivery Fear Scale (DFS). The items are scored based on a 10-point Likert scale ranging from 1 (strongly disagree) to 10 (strongly agree) and the total score range is between 10 and 100, as the higher the score, the greater the fear [12]. The reliability of the Persian version of the tool is good and the Cronbach’s alpha coefficient of DFS constructs in Iran has been calculated to be 0.77 [13]. The intensity of postpartum childbirth fear was measured using the Wijma Delivery Expectancy/Experience Questionnaire B (WDEQVersion B), developed by Wijma et al. Mothers denote their personal feelings and knowledge based on a 6-point Likert scale ranging from 0 to 5. In general, the score is obtained from the sum of the scores of all items and the total range of scores is between 0 -165 [14]. The validity and reliability of the questionnaire in Iran has been confirmed by Mortazavi et al. The Cronbach’s alpha coefficient of W DEQ Version B has been calculated to be 0.914 [15]. EPDS with 10-item was employed to assess postpartum depression based on 4-point Likert scale ranging from 0 to 3 with the total score range of 0–30 [16]. . The validity and reliability of this questionnaire in Iran has been confirmed in the study of Montazeri. The reliability of the questionnaire has been calculated to be 0.77 and 0.8 using the Cronbach’s alpha and experimental methods, respectively [17]. The pain-sensitive scale was applied to measure pain and its information has validity and reliability. The patient rates her current level of pain on 10 cm line from 0 (no pain) to 10 (the most intense pain imaginable) [18]. . The validity of this tool has already been assessed and its correlation coefficient has been calculated to be 0.97 (95% CI: 0.96 to 0.98) [19].

### Data analysis

The data were analyzed using the SPSS software, Version 24.0 (IBM Inc., Armonk, NY, USA). The descriptive statistics was applied to report quantitative and qualitative variables, including mean (standard deviation) and frequency (percent). Independent t-test and ANCOVA were used to compare pre and post mean score of outcomes in present study. A p-value less than 0.05 was considered as statistically significant.

## Results

The mean age (SD) of the participants in the non-pharmacological and pharmacological analgesia groups was 27.03 (5.30) and 27.27 (5.90) years, respectively. Table 1 shows other socio-demographic characteristics of the participants. After the intervention, the mean (SD) score of the fear during labor was 48.42 (9.49) in the non-pharmacological analgesia group and 65.06 (17.14) in the pharmacological analgesia group, indicating a statistically significant difference between the two groups based on the ANCOVA test (MD: -6.33, 95% CI: -10.19 to -3.12, *p* = 0.04). The mean (SD) score of postpartum FOC was lower in the non-pharmacological analgesia group according to ANCOVA test (MD: -21.89, 95% CI: -35.12 to -8.66, *p* = 0.002). After the intervention, the mean (SD) score of postpartum depression was significantly lower in the non-pharmacological analgesia group based on the ANCOVA test (MD: -1.93; 95% CI: -3.48 to -0.37, *p* = 0.01) (Table 2).

**Table 1:** Socio-demographic characteristics of the participants

Characteristics	Non-drug analgesia(*n* = 66)	drug analgesia(*n* = 66)	P-Value
	Mean (SD^b^)	Mean (SD^b^)	
**Age** (Year)	27.03(5.30)	27.27(5.91)	0.33*
**Education**			0.02*
Under diploma	21(63.6)	9(27.3)	
Diploma	9(27.3)	23(69.7)	
University	3(9.1)	1(3.0)	
**Body mass index** (kg/m2 )	25.37(3.57)	24.40(2.69)	0.35*
Job			0.50*
Housewife	32(97.0)	32(97.0)	
employed	1(3.0)	1(3.0)	
**Sufficiency of income for expenses**			0.76†
Insufficient	4(12.1)	7(21.2)	
Somewhat sufficient	29(87.9)	26(78.8)	
Completely sufficient	0(0.0)	0(0.0)	
**fetal sex**			0.85†
Male	19(57.6)	22(66.7)	
Female	14(42.4)	11(33.3)	
**Pharmacologic pain management**			0.54†
Did not receive	31(93.9)	33(100)	
Received(Hyosin)	2(6.1)	0(0.0)	
**Induction or augmentation of labor by Oxytocin**			0.20†
YES	11(33.3)	11(33.3)	
NO	22(66.7)	22(66.7)	
**The birth agent**			0.46†
Women’s resident	22(66.7)	24(72.7)	
midwife	11(33.3)	9(27.3)	
**Nipple stimulation**			0.45†
YES	9(27.3)	6(18.2)	
NO	24(72.7)	27(81.8)	
**Birth weight (g)**	50.66(1.61)	50.36(1.63)	0.49*
**height of the baby**	3306.06(343.63)	3403.63(422.60)	0.88*

* Chi-Square test; † Independent t-test

**Table 2:** Comparison fear of childbirth and postpartum depression among the study groups

Variable	Non-drug pain relief (*n* = 33)	drug pain relief(*n* = 33)	MD (95% CI)a	P-Value
	Mean (SD^b^)	Mean (SD^b^)		
**Fear of childbirth during labor before intervention** Score range:( 10 to 100)	54.06(7.73)	53.60(11.87)	-1.04(-2.87 to-0.81)	0.09*
**Fear of childbirth during labor after intervention** Score range:( 10 to 100)	48.42(9.49)	65.06(17.14)	-6.33(-10.19 to-3.12)	0.04†
**Postpartum Fear of Childbirth** Score range:( 0 to 165)	45.24(10.21)	68.30(11.18)	-21.89(-35.12to-8.66)	0.002†
**Depression before intervention** (Score range: 0 to 30)	4.84(3.72)	4.69(3.29)	0.15(1.88to-1.57)	0.86*
**Postpartum depression** (Score range: 0 to 30)	3.78(2.11)	5.09(2.95)	-1.93(-3.48to-0.37)	0.01†

*Independent t-test; † ANCOVA; a Mean Difference (95% Confidence Interval) with controlling the effect of base score, stratified factor and other confounding factors such as induction with oxytocin and length of labor; b Standard Deviation

The mean (SD) of the labor pain score was lower in the pharmacological analgesia group and illustrating a statistically significant difference between the two groups based on the ANCOVA test (MD: -1.89; 95% CI: -3.06 to -0.89, *p* = 0.03). The mean (SD) of the Apgar score of the newborn in the first minute was 8.7 (0.5) in the non-pharmacological analgesia group and 8.4 (0.4) in the pharmacological analgesia group. The data analysis based on the independent t-test demonstrated no statistically significant difference in the mean Apgar score of the newborn between the two groups. Two women in the pharmacological group (6.1%) underwent CS due to the cessation of labor progress for more than 2-hour. Based on the chi-square test, there was no statistically significant difference between the two groups in terms of delivery mode (*p* = 0.08) (Table 3).

**Table 3:** Comparison of delivery outcomes (maternal and neonatal) among the study groups

Variable	Non-drug pain relief (*n* = 33)	drug pain relief(*n* = 33)	MD (95% CI)^a^	P-Value
	Mean (SD^b^)	Mean (SD^b^)		
**Pain intensity before intervention** (Score range: 0 to 10)	5.60(2.58)	6.51(1.76)	-0.90(-1.99 to 0.18)	0.10*
**Pain intensity after intervention** (Score range: 0 to 10)	6.60(1.44)	4.72(1.48)	-1.89(-3.06 to-0.8)	0.03†
**Apgar score of the first minute**	8.7(0.5)	8.4(0.4)	0.3(-0.6to0.2)	1.0*
**Apgar score of the five minute**	8.8(0.6)	8.6(0.6)	0.2(-0.5to0.1)	1.0*
**Mode of Delivery in all participants**				0.08^¥^
Normal vaginal delivery (spontaneous) (NVD)	33(100)	31(94)		
Emergency cesarean section	0(0.0)	2(6.1)		

*Independent t-test; a Mean Difference (95% Confidence Interval); b Standard Deviation; † ANCOVA; a Mean Difference (95% Confidence Interval) with controlling the effect of base score, stratified factor and other confounding factors such as induction with oxytocin and length of labor; ¥ Chi-square

## Discussion

Based on the findings, non-pharmacological childbirth pain relief methods (back massage, lukewarm water abdominal shower, pressure on the sacrum, breathing techniques, and upright positions) can help reduce the FOC and postpartum depression. In the present study, the mean score of fear during labor and after delivery and postpartum depression in non-pharmacological analgesia group was lower than that in the group receiving pharmacological analgesia with remifentanil. Given that no study was found regarding the effect of pharmacological and non-pharmacological methods on the FOC, the results of the studies that separately investigated the effect of these approaches on the FOC were used to compare with the findings of the present study.

Logtenberg et al. (2018) compared FOC in groups receiving pharmacological analgesia with remifentanil and epidural anesthesia among 409 low-risk pregnant women and reported that women receiving pharmacological analgesia with continuous epidural infusion and remifentanil experienced more FOC during the postpartum period. On the other hand, women with high labor fear requested more pain relief during labor. Although the increase in request for pain relief was not statistically significant [20], the findings of the aforementioned study are consistent with the results of the present study. It seems that pharmacological analgesia does not reduce FOC and other factors are involved in FOC, which should be taken into consideration during intervention.

In a cross-sectional study, Deng et al. (2012) compared the FOC, intensity of labor pain, and analgesia during labor among nulliparous and multiparous women and reported higher FOC in the group receiving epidural anesthesia [21], which is in line with the results of the present study.

Accordingly, applying pharmaceutical methods of labor pain relief causes the parturient women to be inactive during the labor phase, which can increase their labor fear. Therefore, employing other non-pharmacological methods of pain relief and the active participation of the mother during labor reduce the labor fear and the possible side effect of pharmaceutical pain relief methods.

Based on the results of the present study, the mean score of the postpartum depression in the non-pharmacological analgesia group was significantly lower than that in the pharmacological analgesia group. Wang et al. (2022) investigated the effect of analgesia techniques, including epidural anesthesia, epidural-spinal anesthesia combination or the use of analgesics, including ketamine and remifentanil, on the psychological outcomes of 200 primiparous women in China (intervention group = 108 and control group = 92). The findings indicated high depression and anxiety score in the vaginal delivery group without analgesia compared to women received analgesia. They reported that the use of pain relief during labor can improve the primiparous women’s negative feelings and self-efficacy and reduce their psychological pressure. The findings are not in line with the findings of the present study, which can be attributed to the type of intervention, as women in the non-pharmacological group received non-pharmacological interventions such as back massage in the present study, while in the study of Wang et al., women in the group of vaginal delivery without analgesia did not receive any intervention that could affect the results of the study.

In a prospective descriptive study, Lim et al. (2020) examined the relationship between labor pain and postpartum depression symptoms among women with epidural analgesia during labor. They revealed that the experience of labor pain even during the postpartum period and how to manage pain and use epidural anesthesia are independently associated with the depression score at 6 weeks after delivery. Further, they reported that epidural anesthesia can lead to a reduction in postpartum pain and depression, which are not consistent with the findings of the present study. This inconsistency may be attributed to the active participation of mothers in childbirth in the non-pharmacologic group in present study.

Further, Orbach-Zinger et al. (2021) in a review study evaluated the relationship between postpartum depression and neuraxial analgesia during labor and failed to find convincing evidence for the relationship in this regard [22]. In a meta-analysis of descriptive studies, Kountanis et al. estimated the correlation between epidural anesthesia during labor and postpartum depression and demonstrated the failure of epidural anesthesia in reducing the possibility of postpartum depression [23]. Therefore, the mother’s activeness in the delivery process and the use of non-pharmacological analgesia methods can be more effective in reducing postpartum depression, which is consistent with the results of the present study. The findings of the present study revealed no statistically significant difference in the mean Apgar score between non-pharmacological analgesia and pharmacological analgesia groups. Consistent with the results of the present study, Murray et al. (2019) presented ten years of experience of using remifentanil in a treatment center and stated that remifentanil has no specific weaknesses compared to other pharmacological analgesia, which in line with the epidural method can lead to acceptable and desirable analgesia during labor. Further, the lack of absorption of remifentanil from the placenta and its ineffectiveness on the baby are regarded as its strengths. For this reason, remifentanil is an appropriate option for having a baby with a high Apgar score in the first and fifth minutes [24].

In addition, the findings of the present study indicated no significant difference between the non-pharmacological analgesia and pharmacological analgesia groups in terms of the mode of delivery. In a cohort study, Buerengen et al. (2022) assessed the relationship between one-to-one midwifery care in the active phase of labor and the use of pain relief during labor among 7,277 women in Norway. They reported that the need for epidural analgesia and CS was lower among primiparous women received one-to-one midwifery care in the active phase of labor compared to those received no one-to-one midwifery care [25], which is in line with the findings of this study.

### Strengths and limitations

This was the first study that compared the effect of non-pharmacological analgesia and pharmacological analgesia with remifentanil on FOC and postpartum depression in Iran. The participants included nulliparous and multiparous women in Tabriz, Iran. Therefore, the results can be generalized to nulliparous and multiparous women living in other similar cities. The use of valid standard tools in Iran was one of the strengths of the present study. Given that the financial limitations in choosing a large statistical community are one of the limitations of the present study, conducting a study with a larger statistical community is recommended. Another limitation was that, impossibility of blinding of the participants due to the nature of the study.

Some strengths of this study included using random selection, allocation method, and allocation concealment technique, using the participants’ native language during counseling sessions, providing the participants with a contact number to answer their questions.

## Conclusion

The findings of the present study indicated a reduction in FOC and postpartum depression among women using non-pharmacological strategies to reduce labor pain and active participation of parturient women in the labor phase compared to women receiving pharmacological analgesia with remifentanil. Therefore, considering the possible side effects of pharmacological methods on the mother and the fetus, non-pharmacological solutions should be used with the active participation and accompaniment of the mother during labor to reduce the FOC.

In addition, maternity-care policy makers should pay more attention to the feelings and concerns of mothers during pregnancy and hold educational sessions about pain relief methods and invite mothers to participate in childbirth care procedures. They should also develop programs to raise the awareness of health care providers about the important role of active birth and maternal accompaniment during childbirth in preventing relevant adverse outcomes and prepare a pleasant childbirth experience for women. Adding active birth counseling sessions for expectant mothers to prenatal care programs can effectively improve the overall health of mothers and infants.

## Data Availability

The datasets used and/or analyzed during the current study are available from the corresponding author upon reasonable request.
